# DNA Methylation in *Cosmc* Promoter Region and Aberrantly Glycosylated IgA1 Associated with Pediatric IgA Nephropathy

**DOI:** 10.1371/journal.pone.0112305

**Published:** 2015-02-03

**Authors:** Qiang Sun, Jianqian Zhang, Nan Zhou, Xiaorong Liu, Ying Shen

**Affiliations:** 1 Department of nephrology, Beijing Children’s Hospital, Capital Medical University, Beijing, China; 2 Institute of Infectious Diseases, Beijing Ditan Hospital, Capital Medical University, Beijing, China; National Center for Scientific Research Demokritos, GREECE

## Abstract

IgA nephropathy (IgAN) is one of the most common glomerular diseases leading to end-stage renal failure. Elevation of aberrantly glycosylated IgA1 is a key feature of it. The expression of the specific molecular chaperone of core1ß1, 3galactosyl transferase (*Cosmc*) is known to be reduced in IgAN. We aimed to investigate whether the methylation of CpG islands of *Cosmc* gene promoter region could act as a possible mechanism responsible for down-regulation of *Cosmc* and related higher secretion of aberrantly glycosylated IgA1in lymphocytes from children with IgA nephropathy. Three groups were included: IgAN children (n = 26), other renal diseases (n = 11) and healthy children (n = 13). B-lymphocytes were isolated and cultured, treated or not with IL-4 or 5-Aza-2’-deoxycytidine (AZA). The levels of DNA methylation of *Cosmc* promotor region were not significantly different between the lymphocytes of the three children populations (*P* = 0.113), but there were significant differences between IgAN lymphocytes and lymphocytes of the other two children populations after IL-4 (*P*<0.0001) or AZA (*P*<0.0001). *Cosmc* mRNA expression was low in IgAN lymphocytes compared to the other two groups (*P*<0.0001). The level of aberrantly glycosylated IgA1 was markedly higher in IgAN group compared to the other groups (*P*<0.0001). After treatment with IL-4, the levels of *Cosmc* DNA methylation and aberrantly glycosylated IgA1 in IgAN lymphocytes were remarkably higher than the other two groups (*P*<0.0001) with more markedly decreased Cosmc mRNA content (*P*<0.0001). After treatment with AZA, the levels in IgAN lymphocytes were decreased, but was still remarkably higher than the other two groups (*P*<0.0001), while Cosmc mRNA content in IgAN lymphocytes were more markedly increased than the other two groups (*P*<0.0001). The alteration of DNA methylation by IL-4 or AZA specifically correlates in IgAN lymphocytes with alterations in *Cosmc* mRNA expression and with the level of aberrantly glycosylated IgA1 (r = −0.948, r = 0. 707). Our results suggested that hypermethylation of *Cosmc* promoter region could be a key mechanism for the reduction of *Cosmc* mRNA expression in IgAN lymphocytes with associated increase in aberrantly glycosylated IgA1.

## Introduction

Immunoglobulin A (IgA) nephropathy (IgAN) is one of the most common glomerulonephritis in the world [1]. It is estimated that 15–20% of patients with IgAN would reach end stage renal disease (ESRD) within 10 years if untreated [2]. Even a late 30-year analysis of 1,012 patients at a single center revealed IgAN is not a benign disease, with about 50% of patients progressing to ESRD within 30 years despite treatment [3].

IgAN is characterized by mesangial IgA-containing immune deposits, often with IgG and/or IgM co-deposits [4, 5]. The pathogenesis of IgAN is unclear [6]. Elevation of aberrantly glycosylated IgA1 is a key feature of IgAN [7–9].The activity of core1 ß1, 3 galactosyl transferase (C1ß3Gal-T, gene: C1GALT1) is closely associated to aberrantly glycosylated IgA1 [10, 11]. Interestingly, biosynthesis of active C1ß3Gal-T requires an endoplasmic reticulum-localized molecular chaperone, *Cosmc* (Core 1 ß1, 3-galatosyltransferase-Specific Molecular Chaperone, gene: C1GALT1C1) [12, 13]. Dysfunctional *Cosmc* results in the formation of inactive C1ß3Gal-T and consequent expression of the Tn antigen, which is associated with several human diseases, including Tn syndrome [14], IgA nephropathy [15], and some tumors [16]. Nowadays, *Cosmc* expression is known to be reduced in IgAN [17–19]. But no *Cosmc* gene mutations were found in IgAN patients [20, 21]. Epigenetic silencing of *Cosmc* with associated reduced *Cosmc* transcription has been reported in human leukocytes expressing Tn antigen [22]. The present study focuses on the Epigenetic silencing of *Cosmc* in IgAN patients.

The aim of our study is to investigate whether the methylation of CpG islands of *Cosmc* gene promoter region could act as a possible mechanism responsible for the down-regulation expression of *Cosmc* and related higher secretion of aberrantly glycosylated IgA1 in lymphocytes from children with IgA nephropathy. This experimental study was performed in vitro on cultured B lymphocytes from children with IgAN, treated or not with interlukin-4 (IL-4) or 5-aza-2’-deoxycytidine (5-Aza-dC, AZA); the IgAN lymphocytes were compared to lymphocytes from children with other renal diseases or from healthy children (S1 Fig.).

## Materials and Methods

### Patients and Specimens

#### IgAN group

Twenty-six children diagnosed with primary IgAN [23,24] were included. Eligible criteria: (1) age 18 or below; (2) hematuria and/or proteinuria; (3) without cardiopulmonary or other system diseases; (4) with informed consent; (5) without acute infection in the last month; (6) before immunosuppressive therapy.

#### Control group with other renal diseases

Samples from Elevenchildren were included. There were mild mesangial proliferative glomerulonephritis, thin basement membrane nephropathy, membranous nephropathy, and Alport syndrome by renal biopsy. Eligible criteria: (1) age 18 or below; (2) hematuria and/or proteinuria; (3) without infection; (4) without systemic diseases; (5) informed consent.

#### Healthy control group

Thirteen healthy children with normal routine urinalysis were included and informed consent was signed.

There was no significant difference in all the three groups on sex and age distribution (Table 1). All patients were admitted to Beijing Children’s Hospital from January 2011 to July 2013, and had anticoagulant blood drawing the next day of renal biopsy. Samples were managed immediately.

**Table 1:** Demographic and clinical features of patients and healthy children participants.

**Groups**	**Cases**	**Male (%)**	**Female (%)**	**Age(y)**	**Proteinuria(mg/d)**
IgA nephropathy	26	19(74.1%)	7(25.9%)	9.93±2.45	991±937
other renal disease	11	8(72.7%)	3(27.3%)	8.80±2.92	751±511
healthy control	13	9(69.2%)	4(30.8%)	12.12±4.52	

Note: There were no significant differences between each two groups. Mean±SD.

We deposit our data to a publicly available database, Biobank For Diseases in Children, Beijing Children’s Hospital, Capital Medical University. This study was approved by the Ethics Committee of Beijing Children’s Hospital, Capital Medical University according to the Declaration of Helsinki.

### Isolation and culture of PBMCs

Peripheral blood mononuclear cells (PBMCs) from patients with IgAN or other renal diseases and healthy controls were isolated from peripheral blood by Ficoll-Hypaque density gradient centrifugation. The B-cell population was enriched from the PBMCs by removal of adherent cells through incubation in a plastic tissue-culture flask for 1 hr at 37°C and removal of T cells by CD3 (panT) Dynabeads, according to the manufacturer’s instructions (Invitrogen Dynal AS, Oslo, NO, USA). An alternative protocol for isolation of IgA surface-positive B cells (CD19 surface positive) included immunofluorescence surface staining followed by cell sorting.

The B cells were cultured in Dulbecco’s modified Eagle’s medium, DMEM supplemented with 10% fetal bovine serum (FBS) and 1% antibiotics (Penicillin-Streptomycin Solution, PSN), and under identical conditions (37°C in humidified 5% CO_2_/95% air) for 48 hours, respectively. For drug treatment, cells were treated with 1 μmol/L AZA (Sigma, St Louis, MO) for 48 hours, changing AZA and medium every 24 hours; cells were treated with 10ng/mL IL-4 for 48 hours; untreated cells were left alone as controls. After cultured 48 hours, cells and culture supernatants were collected.

### RNA Extraction and Determination of Cosmc mRNA by RT-PCR

RNA was extracted from cells using an RNA isolation reagent (TRIzol; Invitrogen, Carlsbad, CA). To prevent DNA contamination, total RNA was treated with RNase-free DNase II (Invitrogen).

The human glyceraldehyde-3-phosphate dehydrogenase gene (GAPDH) was used as an internal control in PCR amplification (GAPDH gene-specific primers: F, 5’-cattaaggagaagctgtgct-3’; R, 5’-gttgaaggtagtttcgtgga-3’). A two-step RT-PCR procedure was performed in all experiments. First, total RNA samples (2 μg per reaction) were reversely transcribed into cDNAs by RT II reverse transcriptase (Invitrogen). Then, the cDNAs were used as templates in PCR with *Cosmc* gene-specific primers (*Cosmc*-F, 5’-atggaaatagaatgcaccac-3’; *Cosmc*-R, 5’-ttggtccaagtctccttta-3’).

The amplification reactions were performed using AmpliTaq Gold DNA polymerase (Applied Biosystems, Foster City, CA) by ABI 7500 Fast Real-Time PCR System (Life Technologies). The PCR was programmed as follows: 2 min at 95°C and 30 to 32 cycles of 30 s at 94°C, 30 s at 58°C, and 30 s at 72°C, with an extension for 10 min at 72°C. Relative quantification of target gene expression in patients compared with normal samples (the ratio of *Cosmc*/GAPDH) was detected and performed with the ΔΔCt method, the relative mRNA fold changes were determined as detailed previously in each sample for six times at last [25]. The normalisation was used the data from the 13 healthy children in this study. The PCR products were analysed by agarose gels (agarose, Biowest, Spain) electrophoresis and the PCR bands were visualized.

### Bisulfite Genomic Sequencing for Cosmc gene Promoter regions

Genomic DNA was purified from cells with Wizard Genomic DNA Purification Kit (Promega, Madison, WI). DNA (2 μg) was bisulfite modified with EZ DNA Methylation Direct Kit (Zymo Research, Orange, CA). Sequence-specific primers (F 5’- TTTTAAGAGAGGGAGGGGAGTTAGG-3’; R 5’- TCCAAACAATAAAACTTCAAATCTCATTC-3’) to amplify the CpG-rich regions of interest were designed using a computer program. The PCR products were amplified, purified, and cloned into a vector (pGEM-T Easy Vector; Promega). Clones were selected through blue-white screening. Finally, the colonies harboring the insert were sequenced in a 96-well plate using the M13 reverse and/or forward primers.

### Methylation-Specific PCR for Cosmc gene Promoter regions

The advanced method Bisulfite sequencing PCR (BSP) for determining methylation levels of gene promoter regions had been used, and all CpG islands of *Cosmc* promoter regions had been detected. The bisulfite-treated DNA was amplified using primers that specifically amplify either the methylated or unmethylated sequence of *Cosmc* promoter, respectively. The PCR was performed for 40 cycles, with annealing temperatures of 58°C for the methylated reaction and 52°C for the unmethylated reaction. The human methylated and unmethylated DNA was used as control to verify the specificity of the primers (QIAGEN, Valencia, CA).

### Measurement of aberrantly glycosylated IgA1

The levels of aberrantly glycosylated IgA1 (IgA1 O-glycosylation) in the supernatant from culture well were determined by Vicia villosa (VV) lectin-binding assay. The 96-well immunoplates (Costar, Cambridge, Mass., USA) were coated with purified human specificity O-glycosylation IgA1 (hinge area α1) monoclonal antibody (Southern Biotechnology Associates, Birmingham, Ala., USA). After centrifugation, the cell culture supernatant was collected. Then, 100 μl of supernatant sample or standard human IgA1 (Calbiochem, La Jolla, Calif., USA) was added to wells and then incubated for 1 h at 37°C. After incubation for 1 h at 37°C with biotinylated VV lectin (Vector Laboratories, UK), washed again, and peroxidase-avidin D (Vector Laboratories), color was developed. After add stop solution to each well, absorbance value at 450 nm was measured. The aberrantly glycosylated IgA1 concentration in samples was detected by interpolation of the respective optical density (OD) into the appropriate standard curve.

### Statistics

Data are expressed as means ± standard deviation (SD). Parametric distributions were analyzed by non-parametric statistics. Comparisons among groups and two groups were evaluated by ANOVA and Mann-Whitney U test. Values of p < 0.05 were regarded as significant. Correlations between two factors were evaluated by pearson correlation analysis. SPSS 13.0 statistical software was used to conduct the analysis.

## Results

### DNA methylation status of CpG islands in the promoter region of Cosmc gene

**Figure 1:**
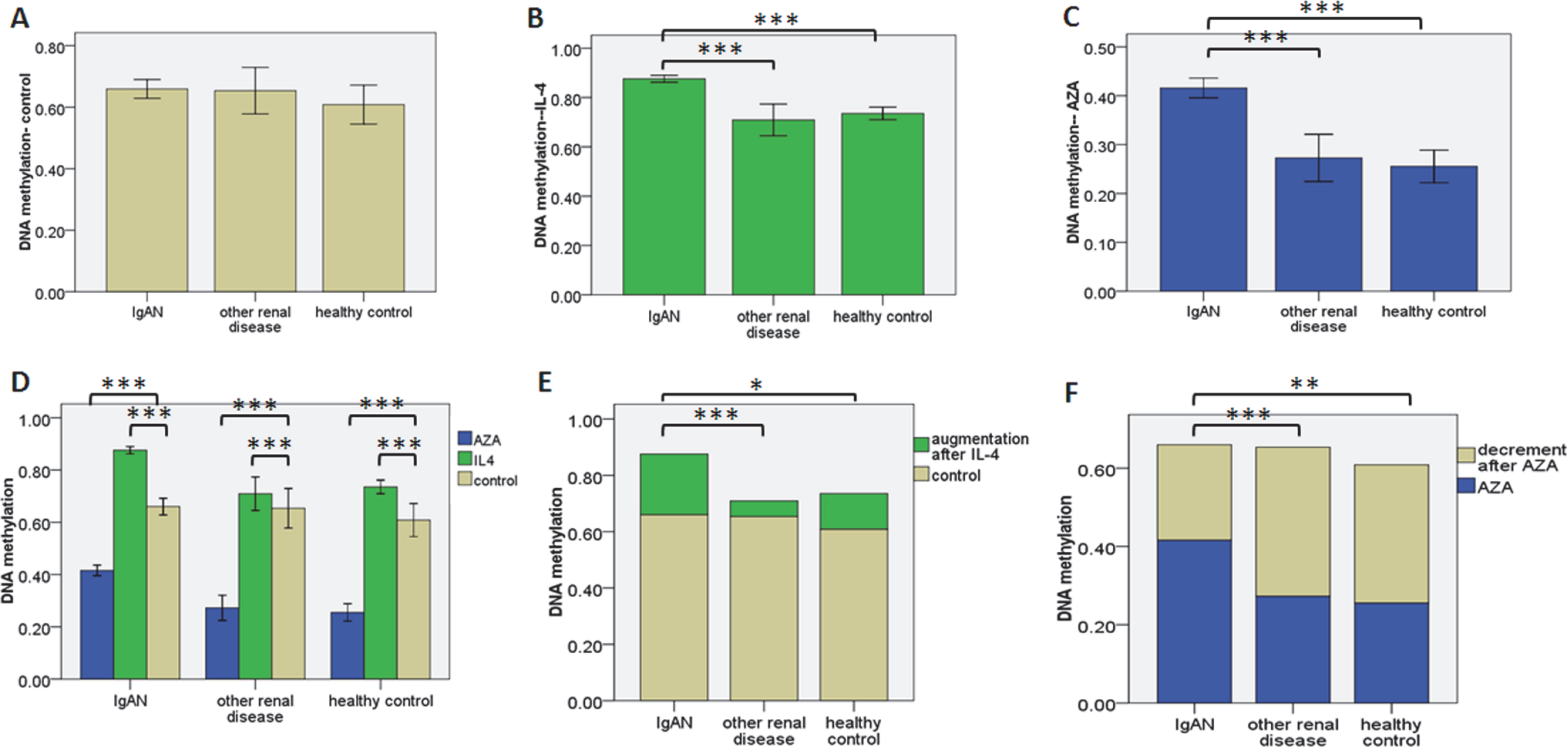
Methylation status of CpG islands in the promoter regions of *Cosmc* gene. DNA methylation levels of CpG islands in the promoter regions of *Cosmc* gene were detected by bisulfite-modification sequencing PCR, and high-throughput quantitative DNA methylation analysis. Samples are B lymphocytes from peripheral blood of the 3 groups (IgAN patients, n = 26; other renal disease patients, n = 11; healthy children participants, n = 13). Error bars represent s.e.m. and * denotes a p-value ≤0.05. * *≤ 0.01. * * *for p-value ≤ 0.001. (a) DNA methylation in 3 groups after 48 hours culturing. (b) DNA methylation in 3 groups after 48 hours culturing with IL-4 (10ng/ml). (c) DNA methylation in 3 groups after 48 hours culturing with AZA (1umol/l). (d) DNA methylation in 3 groups treated or not (beige) with IL-4 (green) or AZA (blue). (e, f) Compared to plain medium, IL-4 or AZA caused the alteration of DNA methylation levels in the other two groups.

The mean levels of DNA methylation of *Cosmc* promotor region, evaluated after 48 hours in vitro culturing with plain medium, were not significantly different between the lymphocytes of the three children populations, *P* = 0.434 (Fig. 1A). With IL-4, *Cosmc* methylation levels in IgAN lymphocytes were higher than that in lymphocytes of the other two children populations (*P* = 1.709E-12, *P* = 4.063E-9, respectively). There was no significant difference between the other two children populations, *P* = 0.094 (Fig. 1B). With AZA, *Cosmc* methylation levels in IgAN lymphocytes decreased, but was still higher than that in the lymphocytes of the other two children populations (*P* = 2.084E-9, *P* = 5.860E-11). There was no significant difference between the latter two groups (*P* = 0.300) (Fig. 1C). For the three lymphocytes groups, compared to no treatment, the levels of *Cosmc* DNA methylation were increased by IL-4 (*P* = 2.770E-23, *P* = 7.969E-5, *P* = 2.071E-4, respectively) and reduced by AZA (*P* = 4.800E-26, *P* = 4.634E-24, *P* = 1.559E-14, respectively) (Fig. 1D). The increment induced by IL-4 was higher in IgAN lymphocytes than in the lymphocytes of the two other children populations (P = 1.157E-4, P = 0.017, respectively). There was no significant difference between the two other groups, P = 0.106 (Fig. 1E). The decrease induced by AZA was lower in IgAN lymphocytes than in the lymphocytes of the two other children populations (P = 4.364E-4, P = 0.003, respectively)There was no significant difference between the latter two groups (*P* = 0.502) (Fig. 1F).

### Detection of Cosmc mRNA expression

**Figure 2:**
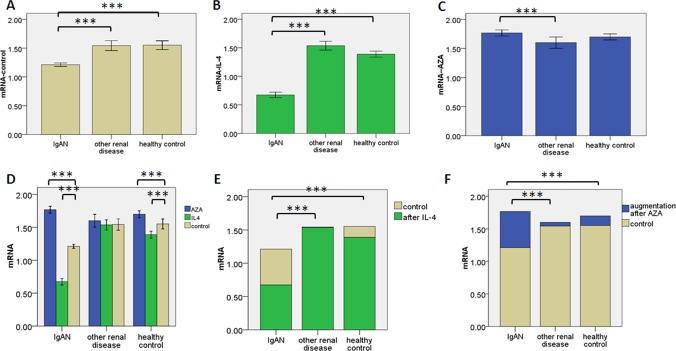
The mRNA expression levels of *Cosmc.* The mRNA expression levels of *Cosmc* by RT-PCR. Samples are B lymphocytes from peripheral blood of the 3 groups (IgAN patients, n = 26; other renal disease patients, n = 11; healthy children participants, n = 13). Error bars represent s.e.m. and * * * for p-value ≤ 0.001. (a) *Cosmc* mRNA expression in 3 groups after 48 hours culturing. (b) *Cosmc* mRNA expression in 3 groups after 48 hours culturing with IL-4 (10 ng/ml). (c) *Cosmc* mRNA expression in 3 groups after 48 hours culturing with AZA (1 umol/l). (d) *Cosmc* mRNA expression in 3 groups treated or not (beige) with IL-4 (green) or AZA (blue). (e, f) Compared to plain medium, IL-4 or AZA caused the alteration of *Cosmc* mRNA levels in the other two groups. The mRNA data in Figure was the relative quantification of the ratio of Cosmc/GAPDH in each sample.

Without treatment, *Cosmc* mRNA levels were lower in IgAN lymphocytes than in the lymphocytes of healthy controls (*P* = 3.340E-13) or other renal diseases *(P* = 1.042E-11). There were no significant difference between the latter two groups (*P* = 0.838) (Fig. 2A). With IL-4, *Cosmc* mRNA levels in IgAN lymphocytes were remarkable lower than lymphocytes of the other two children populations (*P* = 5.993E-27, *P* = 1.710E-22).There was significant difference between the other two children populations, *P* = 0.002 (Fig. 2B). With AZA, there were no significant differences between the lymphocytes of IgAN and healthy controls (*P* = 0.107). While the levels in IgAN lymphocytes were higher than that in the lymphocyte of other renal diseases, *P* = 4.587E-4. There was no significant difference between the lymphocytes of healthy controls and other renal diseases, *P* = 0.058 (Fig. 2C). For the lymphocytes of IgAN and healthy controls, compared to no treatment, *Cosmc* mRNA levels were decreased by IL-4 (*P* = 1.104E-29, *P* = 2.066E-4) and increased by AZA (*P* = 1.540E-30, *P* = 0.001) (Fig. 2D). In the lymphocytes of other renal diseases, compared to no treatment, there were no significant differences with IL-4 (*P* = 0.312) or AZA (*P* = 0.910) (Fig. 2D). The decrease induced by IL-4 was higher in IgAN lymphocytes than in the lymphocytes of the two other children populations (P = 1.808E-16, P = 3.529E-12) (Fig. 2E). The increment induced by AZA was higher in IgAN lymphocytes than in the lymphocytes of the two other children populations (P = 2.759E-4, P = 1.837E-17).There was significant difference between the latter two groups (*P* = 0.035) (Fig. 2F).

### Detection of aberrantly glycosylated IgA1

**Figure 3:**
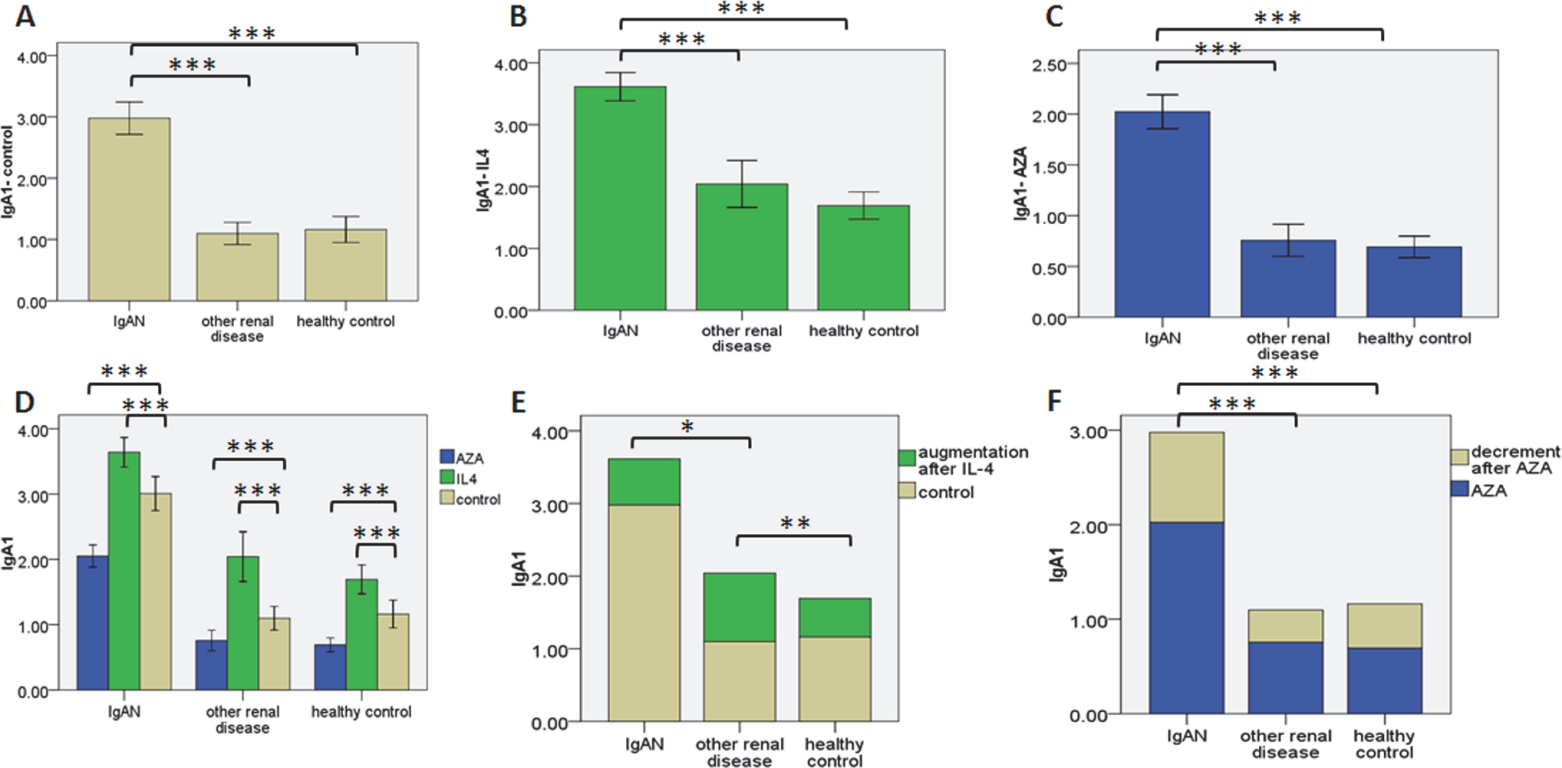
The levels of aberrantly glycosylated IgA1. The levels of aberrantly glycosylated IgA1 in culture supernatant of B lymphocytes were detected by ELISA. B lymphocytes were from peripheral blood of the 3 groups (IgAN patients, n = 26; other renal disease patients, n = 11; healthy children participants, n = 13). Error bars represent s.e.m. and * denotes a p-value ≤0.05. * *≤ 0.01. * * * for p-value ≤ 0.001. (a) The levels of aberrantly glycosylated IgA1 in 3 groups after 48 hours culturing. (b) The levels of aberrantly glycosylated IgA1 in 3 groups after 48 hours culturing with IL-4(10ng/ml). (c) The levels of aberrantly glycosylated IgA1 in 3 groups after 48 hours culturing with AZA (1 umol/l). (d) The levels of aberrantly glycosylated IgA1 in 3 groups treated or not (beige) with IL-4 (green) or AZA (blue). (e, f) Compared to plain medium, IL-4 or AZA caused the alteration of aberrantly glycosylated IgA1 levels in the other two groups.

Without treatment, levels of aberrantly glycosylated IgA1 were remarkable higher in IgAN lymphocytes than the lymphocytes of healthy controls (*P* = 3.310E-13) or other renal diseases (*P* = 1.595E-13). There was no significant difference between the latter two groups (*P* = 0.766) (Fig. 3A). With IL-4, the level of aberrantly glycosylated IgA1 in IgAN lymphocytes increased and was still higher in IgAN lymphocytes than in the lymphocytes of the two other children populations (P = 7.436E-11, P = 2.360E-14). There was no significant difference between the other two groups (*P* = 0.109) (Fig. 3B). With AZA, the levels of aberrantly glycosylated IgA1 in IgAN lymphocytes were remarkably higher than lymphocytes of the other two children populations (*P* = 5.330E-14, *P* = 1.340E-15). There was no significant difference between the latter two groups (*P* = 0.637) (Fig. 3C). For the three lymphocytes groups, compared to no treatment, the levels of aberrantly glycosylated IgA1 were increased by IL-4 (*P* = 8.753E-5, *P* = 2.999E-6, *P* = 0.047, respectively) and reduced by AZA (*P* = 1.956E-8, *P* = 4.446E-4, *P* = 1.041E-4, respectively) (Fig. 3D). The increment induced by IL-4 in lymphocytes of other renal diseases was more than that in the lymphocytes of healthy controls (*P* = 0.007) or IgAN *(P* = 0.022) (Fig. 3E). The decrement induced by AZA in IgAN lymphocytes was more than that in lymphocytes of the other two children populations (*P* = 3.286E-5,*P* = 3.692E-4). There were no significant difference between the latter two groups (*P* = 0.400) (Fig. 3F).

### Analysis of correlation

#### Correlation between DNA methylation in *Cosmc* promoter regions and *Cosmc* mRNA expression

**Figure 4:**
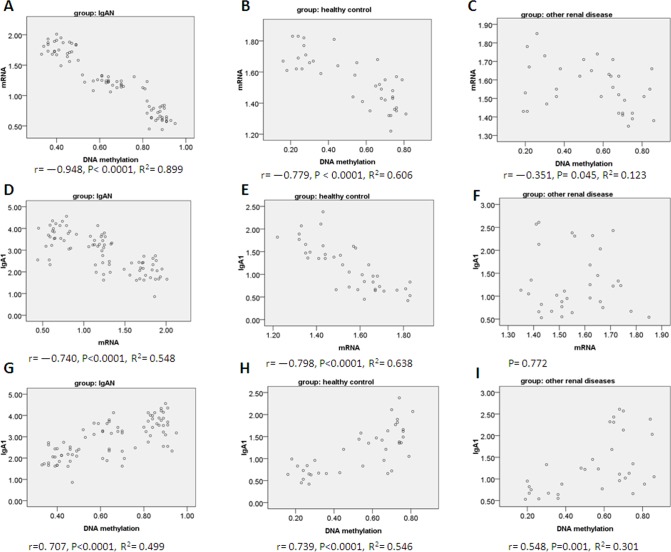
Analysis of correlation. Pearson correlations in 3 groups (IgAN group, n = 78; other renal disease group, n = 33; healthy children group, n = 39) between (A, B, C) Cosmc DNA methylation and Cosmc mRNA expression; (D,E,F) between aberrantly glycosylated IgA1 and Cosmc mRNA expression; (G,H,I) between aberrantly glycosylated IgA1 and Cosmc DNA methylation.

There was a negative linear correlation in IgAN lymphocytes, lymphocytes of healthy controls and other renal diseases (Fig. 4A, r = −0.948, *P* = 3.795E-41, R^2^ = 0.899; Fig. 4B, r = −0.779, *P* = 5.304E-9, R^2^ = 0.606; Fig. 4C, r = −0.351, *P* = 0.045, R^2^ = 0.123).

#### Correlation between aberrantly glycosylated IgA1 and *Cosmc* mRNA expression.

There was a negative linear correlation in IgAN lymphocytes and lymphocytes of healthy controls (Fig. 4D, r = −0.740, *P* = 2.818E-15, R^2^ = 0.548; Fig. 4E, r = −0.798, *P* = 1.129E-9, R^2^ = 0.638). There was no correlation in lymphocytes of other renal diseases (Fig. 4F, *P* = 0.772).

#### Correlation between aberrantly glycosylated IgA1 and DNA methylation in *Cosmc* promoter regions

There was linear positive correlation in the lymphocytes of all the three populations (Fig. 4G, r = 0. 707, *P* = 1.684E-13, R^2^ = 0.499; Fig. 4H, r = 0.739, *P* = 7.711E-8, R^2^ = 0.546; Fig. 4I, r = 0.548, *P* = 0.001, R^2^ = 0.301).

## Discussion

*Cosmc* is the specific molecular chaperone in the endoplasmic reticulum for T-synthase, a Golgi ß1, 3-galactosyltransferase that generates the core 1 O-glycan, Galß1,3GalNAcɑ-Ser/Thr, in glycoproteins. Dysfunctional *Cosmc* leads to the formation of inactive T-synthase and consequent expression of the Tn antigen, which is associated with Tn syndrome and IgAN. It is reported that the *Cosmc* promoter in B cells from a patient with Tn antigen-positive leukocytes is methylated and AZA treatment restores *Cosmc* transcription and normal O-glycans [22]. In this work, we investigated whether the methylation of CpG islands of *Cosmc* gene promoter could act as a possible mechanism responsible for the down regulation of *Cosmc* mRNA and related higher secretion of aberrantly glycosylated IgA1 in lymphocytes from children with IgAN.

As expected (consistent with IgAN pathology), the level of aberrantly glycosylated IgA1 was markedly higher in supernatants of lymphocytes from IgAN children compared to the other groups. The levels of aberrantly glycosylated IgA1 were increased by IL-4 and reduced by AZA for the three lymphocytes groups; interestingly, reduction with AZA was more important in the IgAN group.

As expected (consistent with IgAN pathology), *Cosmc* mRNA expression was low in IgAN lymphocytes compared to the two other groups. IL-4 treatment enhanced the difference between the IgAN group and the others by more markedly decreasing *Cosmc* mRNA content in IgAN lymphocytes. Conversely, *Cosmc* mRNA content was regained after AZA treatment, markedly for IgAN lymphocytes and moderately for healthy lymphocytes, so that the differences in mRNA levels were markedly reduced between the three populations after AZA treatment.

In this study, *in vitro* IL-4 treatment revealed differences between the three lymphocytes populations; it very significantly increased *Cosmc* promoter methylation in IgAN lymphocytes whereas the increase was moderate in control healthy lymphocytes and no significant for lymphocytes of patients with other renal diseases. These results are consistent with conclusion of Yamada K et al. [17], which showed that IL-4 reduced the expression of C1GALT1 in IgA1-positive human B-cell line. Since C1ß3Gal-T (gene: C1GALT1) plays a role with its specific molecular chaperone—*Cosmc* [12], we suppose the reduced expression of *Cosmc* to be the cause of the reduced the expression of C1ß3Gal-T. Further experiments are still needed to confirm it.

He et al [6] reported that the IL-4/STAT6 signaling pathway was highly activated in all tonsil tissues of IgAN patients. It showed that activation of the IL-4/STAT6 signaling pathway may have a crucial role in secretion of aberrantly glycosylated IgA1. Also, we noticed that in clinic, some onset of IgAN in children patients were induced by infections. Inflammation could produce cytokines (e.g.IL-4) [26], so the decreased levels of *Cosmc* mRNA with the increased levels of aberrantly glycosylated IgA1 and the methylation of *Cosmc* promoter region caused by IL-4 may be one of the mechanisms of why the disease could be induced by infections.

Conversely, the results showed that AZA treatment reduced DNA methylation in lymphocytes of the three populations; reduced the levels of aberrantly glycosylated IgA1 and increased *Cosmc* mRNA markedly for IgAN lymphocytes.

As DNA methyltransferase 1 (DNMT1) inhibitor [27,28], AZA can inhibit DNA methylation. Our results showed that *Cosmc* mRNA content was regained and the levels of aberrantly glycosylated IgA1 were reduced after AZA treatment. We concluded that AZA inhibited the DNA methylation of *Cosmc* promoter region and partially restored the activation and function of inhibited *Cosmc* gene(C1GALT1C1). Interestingly, reduction or the increase with AZA was more important in the IgAN group. This suggests there are higher levels of *Cosmc* DNA methylation in IgAN lymphocytes than the other two groups.

We presume DNA methylation of *Cosmc* gene (C1GALT1C1) could act as a possible mechanism responsible for the down regulation of *Cosmc* and related higher secretion of aberrantly glycosylated IgA1 in lymphocytes from children with IgAN. Also, the results of the correlation analysis support it. The correlation between *Cosmc* DNA methylation and *Cosmc* mRNA expression was specifically very strongly negative for the IgAN lymphocytes. This suggests that *Cosmc* DNA methylation could be a major determinant of *Cosmc* mRNA expression especially in the IgAN group, but not in the other diseases group. For the other diseases lymphocytes, the correlation between *Cosmc* DNA methylation and *Cosmc* mRNA expression was very poor, suggesting that factors different from *Cosmc* DNA methylation may regulate *Cosmc* mRNA expression in the other diseases. Moreover, in the IgAN group only, concomitant aberrantly glycosylated IgA1 positively correlated with DNA methylation and negatively correlated with *Cosmc* mRNA. For the lymphocytes of patients with other renal diseases, the correlation between aberrantly glycosylated IgA1 levels and *Cosm*c DNA methylation was poor.

However, unexpected, the mean levels of DNA methylation of *Cosmc* promotor region, evaluated after 48 hours *in vitro* culturing with plain medium, were not significantly different between the lymphocytes of the three children populations. This finding seems to be not consistent with the hypothesis that increased *Cosmc* DNA methylation could cause reduced *Cosmc* expression in IgAN. The reason may lies in the following three points. First, the sample may be not large enough. In this study, we only include 50 voluntary participants. The three groups may be not representative of the entire populations. What’s more, this experimental study was performed *in vitro* on cultured B lymphocytes from children with IgAN, other renal disease and healthy children. Maybe an existing native hypermethylation (potentially present *in vivo*) be reversed by the 48 hours lymphocytes culturing in plain medium. Further *in viv*o evaluations on large populations are needed. At last, the potential key factors that influencing *Cosmc* mRNA expression through DNA methylation might be not the levels but the alteration of the status of *Cosmc* gene methylation. Hypermethylation at some key site of CpG islands of *Cosmc* gene promoter region might play a decisive role in altering the status of *Cosmc* gene DNA, which consequently altering the mRNA expression of *Cosmc*. In the present study, we tested DNA methylation levels at every site of CpG islands in *Cosmc* gene promoter and analyzed the total incidence of DNA methylation. Furthermore, we are going to focus on each site of CpG islands to analyze the status of methylation.

We noticed that in this study, with AZA treatment, the reduction of aberrantly glycosylated IgA1 and the increase of *Cosmc* mRNA expression were more marked in IgAN lymphocytes. While, the reduction of DNA methylation being less marked in IgAN lymphocytes, so that the level of *Cosmc* DNA methylation after AZA remained higher in IgAN lymphocytes than the other two groups. We consider the cause may lies in the following two points. Firstly, the level of Cosmc DNA hypermethylation in IgAN might be stable and not as easily being reversed as in the other two groups. Secondly, since AZA is a nonspecific DNA methyltransferase inhibitor, there is the possibility that not only *Cosmc* (gene: C1GALT1C1), but also other genes, for example, C1GALT1, whose methylation levels were influenced by methyltransferase inhibitor, and accordingly led to the alteration of mRNA expression of *Cosmc*. Till now, no researches on the evaluation of the DNA methylation of C1GALT1 or other genes potentially involved in aberrant glycosylation of IgA1 have been reported. Further studies may be needed.

Limitations of the study: Besides the relatively small size and lack of Researches *in vivo*, correlations in this work suggest a functional relationship between *Cosmc* DNA methylation, *Cosmc* mRNA and the secretion of aberrantly glycosylated IgA1, specifically in lymphocytes of patients with IgAN. However, associations are not demonstrations of causality. Also, since it’s largely univariate analysis, there might be other co-factors influencing *Cosmc* mRNA and aberrant glycosylation of IgA1. Recently, it is reported that the expression of miR-148b also has a positive correlation with aberrantly glycosylated IgA1 but has no correlation with Cosmc [29]. We will have further studies.

In general, this study showed that experimental modifications of DNA methylation revealed a specific behavior of the lymphocytes from IgAN children, compared to lymphocytes of the patients with other diseases or healthy control, concerning methylation of *Cosmc* promoter and associated *Cosmc* mRNA expression. IL-4 induced higher hypermethylation of the *Cosmc* promoter region whereas the demethylating agent AZA was less efficient on *Cosmc* methylation in the IgAN lymphocytes than in the other two groups. By the same time, IL-4 further reduced *Cosmc* mRNA and increased aberrantly glycosylated IgA1 levels in IgAN lymphocytes; AZA restored *Cosmc* mRNA to a level even slightly higher than in cells of healthy control, and leaded to the related lower secretion of aberrantly glycosylated IgA1 in lymphocytes from children with IgAN.

The results suggest that hypermethylation of *Cosmc* gene promoter could be a key mechanism for the reduction of *Cosmc* mRNA expression in lymphocytes of patients with IgAN and associated to the increased level of aberrantly glycosylated IgA1. Further *in vivo* studies with larger samples are still needed in the future.

## Supporting Information

S1 Fig (DOCX)

S1 Table (XLS)
