# Supplementary material for: DNA Methylation in Cosmc Promoter Region and Aberrantly Glycosylated IgA1 Associated with Pediatric IgA Nephropathy
Source: PLoS One. 2015 Feb 3;10(2):e0112305. doi: 10.1371/journal.pone.0112305 (PMC4315396; doi:10.1371/journal.pone.0112305)
Supplement: S1 Fig — (DOCX) [file pone.0112305.s001.docx]

**Flow diagram**

To detect

Cosmc DNA methylation

aberrantly glycosylated IgA1

Cosmc mRNA
